# General Observations on the Nature and Cure of Hydrophobia

**Published:** 1809-02

**Authors:** James Wood


### *To the Editors of the Medical and Physical Journal.*

GENTLEMEN,

SOME years ago, as will appear from the date of the annexed paper, with the view to make up my mind on the nature and cure of Hydrophobia, in the event of my meeting with such a case, I consulted almost every author on the subject, and collected all the information in my power; and gave the result in the following paper to the Medical Society of this town. As the subject appears at present to be an interesting one, in the metropolis especially; and as the labour I took may save some others a similar task, by transmitting it to you for insertion in your Journal, I have therefore determined to do so, if you think proper, and I will be glad if it should in any way be productive of public benefit.

I am, &c.

JAMES WOOD, M. D.

---

### GENERAL OBSERVATIONS ON THE NATURE AND CURE OF HYDROPHOBIA.

Addressed to the Medical Society of Newcastle.

AFTER collecting all the information that could be procured on the nature and cure of that diseased state of the human body, occasioned by the poison of a rabid animal, I found that I could not lay the same before the Society, agreeably to my first intention, but in a very voluminous form, which my leisure, in the time limited, would not permit me to do, and which indeed appeared to me superfluous too, when such full and able views have been already given of the disease by many eminent men, particularly among the moderns, by Drs. Hamilton, Maese, Bardsley, Vaughan, Heysham, Arnold, Hunter, Percival, Haygarth, and others. To endeavour to attain the end, therefore, which first induced me to call the attention of the Society to this disease, I will attempt to comprize all the information I have obtained, in the form of general inferences and conclusions, which can be supported by the rules of just induction, from experiments, from well-authenticated phenomena, and from analogy; and where such are wanting, I will adopt conclusions supported by the the opinion of the majority in any question of doubt or point of dispute. I need not I dare say repeat, that the motives I have in attempting this, are, that truth may be separated from fable and superstition, and that we may be enabled to form a *quick decision,* and be impressed with a conviction of the importance of adopting, and firmly persevering in, the most approved plan of prevention and cure; the unsteady medley practice hitherto generally pursued, appearing to me to be the chief cause of the malady continuing the opprobrium medicinæ.

1. The disease known by the name of Hydrophobia, and occasioned by the poison of a rabid animal, appears to have existed in the most ancient times; it seems to be coeval with the dog itself, which animal has existed in all ages. The rage of the mad dog is alluded to metaphorically by Homer. It is not mentioned by Hippocrates; it therefore appears probable that it did not exist in his time near the country in which he lived. It was certainly known in the time of Asclepiades, and is mentioned by almost every writer since his time. Some countries have continued almost entirely exempt from the disease. In South America, for instance, it was never known; nor was it known in Jamaica for fifty years before 1783.

2. The saliva of any rabid animal will communicate this disease; but there is reason to believe that there is no instance of its being returned from the human species to the quadruped, the attempt having been made by inoculation. We may also venture to conclude that the disease has never been communicated by the saliva of a human person. The saliva of quadrupeds only seems capable of assimilating with the poison; and of these, the genera, canis and felis, are the most remarkable, though horses, mules, oxen, deer, sheep, and swine may be included in the number. How far domestic poultry and other fowls may be included, appears uncertain.

3. There are strong presumptive proofs that rabies originates spontaneously in many quadrupeds; and the proofs are nearly positive that it arises spontaneously in the dog. *Carnivorous* animals seem most, if not only, liable to it as a spontaneous disease.

4. There seems sufficient reason to believe, that a train of symptoms, resembling those occasioned by specific contagion of rabies, have taken place in the human body as the effect of causes, producing a similar diseased action, but independent of any specific contagion being applied, or by that action generated in the body; and that such such symptoms vary only in degree from those produced by the canine poison.*

5. The poison of a rabid animal may produce the disease through different mediums, but contact seems always necessary; there is reason to fear that contact alone, without any wound, has occasioned the disease, when the poison has been applied to those parts where the cuticle is thin, such as the lips; it has been communicated by clothes torn by rabid animals and applied to the lips; it has also been conveyed by a weapon used in killing a rabid animal. There are no proofs that any of the secretions of a rabid animal but the saliva can excite the disease; any infection having been conveyed through the medium of the breath seems also groundless.

6. A large proportion of such persons as have been really wounded by the bite of a rabid animal are never affected with the disease. Mr. Hunter mentions an instance of twenty persons being bitten by the same dog, and only one had any disease in consequence; there are several instances of only one of twelve or fifteen persons bitten being seized with the disease.

7. Here may be noticed the general established fact, that different persons are not alike predisposed to be acted on by the same contagion, as well as that the predisposition to receive contagion varies in the same person at different periods. Dr. James says, that some persons are positively exempt from any danger from the canine virus, however applied. We know some persons never take the small-pox; some seem insensible to the venereal virus; the dog and the ass, by the experiments of Mr. Hunter, cannot be acted on by the venereal virus.

8. The disease that takes place in consequence of the canine poison having been applied by inoculation or otherwise, has appeared at very different periods after the application of it to the body. The age and state of the body; the season of the year; the climate; the degree of virulence of the poison, may be so many causes of this variation, and some of these circumstances may serve also to explain the nature of the predisposing causes. Women and

* Indeed, we may almost venture to conclude, that even when the disease is excited by the poison of a rabid animal, that there is no such diseased action takes place in the salivary glands in the human species as certainly does in the canine race, and other animals, assimilating that secretion in such a manner as to make it capable of exciting the disease in any other animal.

and children are observed to be not only most susceptible of the infection, but also in them the disease appears always at an early period after the application of the poison. The depressing passions have had a considerable influence in occasioning the predisposition to this disease, which has in many instances soon appeared after their debilitating effects; there are other instances too, where other causes producing *debility* have predisposed the system to the action of the virus, which, in many, seemed to have remained passive, until *that state* took place in a remarkable degree. There are no well authenticated cases of its lying dormant longer than eleven or twelve months, excepting one case, in which there is good reason to suppose it did not appear for twenty months after the accident. Very few have exceeded five or six months; and the common time of its appearance is from twenty to forty days after the bite; we may therefore consider any person safe when twelve months have elapsed without any symptom appearing.

9. It seems doubtful whether the poison enters the system by the absorbents, or acts immediately on the nerves; this is a much disputed question, and there are even grounds of analogy for argument in support of both opinions; the question, however important, as leading to a just theory, it does not seem requisite at present to decide, as the supporters of both opinions *do* not differ in the smallest degree in the means to be used for the prevention or cure.

10. In most instances the wound from the bite of a rabid animal heals the same as any other wound made in the same way, by the same animal, in health. Dr. James says, that he considers it as a *certain symptom of the presence of the poison,* if the wound remains any time *covered with a scab.*

11. The part that had been injured generally becomes painful, sometimes inflames, and swells prior to the appearance of the dreadful symptoms of the disease, which soon follow; the pain is commonly described as shooting from the part that had been wounded, in the course of the lymphatics towards the heart, or where they unite with the sanguiferous system; sometimes it shoots with great agony from the part, and fixes in the throat; there are however two or three well authenticated cases of the other symptoms taking place, without any complaint of pain from the part that received the poison, or any reference having been made to it by the patient; with, however, the prelude of pain in several, the more constant symptoms of the disease begin in the following order; they have been divided into three stages.

12. First stage, lassitude, inactivity, sensation of weight, particularly about the precordia, subsultus tendinum; sighing, great dejectedness, with a desire of solitude; restlessness with timidity, often prevails; the sleep (if any, for there is often no sleep) very disturbed, with terrifying dreams; external senses very acute; the common air intolerable; the least noise disturbs; the light, as well as strong colours, water, or any transparent body, very offensive; tongue dry; great thirst; eyes quick and penetrating, with something not easily expressed in the countenance; the recumbent posture disagreeable; the pulse unsteady, changing frequently, often intermitting; sickness; vomiting.

Second stage. Pain (if there has been any) in the bitten part now vanishes; the muscles of the gula, as soon as water or other liquids touch them, become convulsed, with the utmost uneasiness in swallowing, and a sense of suffocation; solids often swallowed with less pain; pain about the cartilago ensiformis; sense of heat in the stomach; palpitations of the heart; titillation in the urethra; urine forcibly expelled by spasms, with often priapism, and emission of semen; copious flow of viscid saliva, which is productive of great distress; swallowing it producing convulsions, and the act of spitting it out difficult, on account of its viscidity.

Third stage. Pain in swallowing with every other symptom increased; solids often still swallowed without much pain; pupil often much dilated, with loss of sight; hands and feet cold; pulse intermitting, weak, and irregular; spasms more frequent and violent; frequent delirium, but commonly attendant on the spasm; in the interval of the paroxysms the mind is commonly collected, and all the mental powers unusually acute and perfect; sometimes in the fits a desire to bite; the strength fails rapidly, and death quickly closes the scene. This event sometimes takes place as if the effect of strangulation; sometimes in the midst of a convulsive fit; very often in a placid manner, without any struggle, and not unfrequently with a smile on the countenance.

13. Death commonly takes place about the third day from the first appearance of the symptoms.

14. Dissections, tho' exhibiting appearances very much alike, do not lead to much explanation of the symptoms; the membrane lining the trachea and œsophagus are sometimes times found slightly inflamed, and sometimes the stomach exhibits the same appearance, and the intestines in different parts; the brain, spinal marrow, and all the viscera are found much dryer than usual; the pericardium without liquor; the lungs stuffed with blood; the heart and arteries very full of blood, and the veins almost empty.

15. There are well authenticated cases of convulsions and death following the bite of a rabid animal, in which no difficulty of swallowing or aversion to liquids took place.

16. The aversion to liquids, with convulsions on swallowing them, has appeared a symptom in other diseases; viz. in gastritis, in hysteria, in epilepsy, in tetanus, in some fevers, and in different species of cynanche.

17. It is very remarkable that the dog, as well as other animals in a rabid state, never experiences that dreadful symptom which so commonly follows, as an effect of his poison, the hydrophobia *; this is contrary to the vulgar opinion.

18. The principal symptoms of the disease produced by the poison of a rabid animal, are explicable on the theory of convulsion, delivered by every pathological writer; the proximate cause of such an affection is generally allowed to be mobility, which seems to consist in increased irritability †; when it shall be discovered what the source of irritability is, we may form more accurate ideas of convulsion; at present, no hypothesis is attempted; facts only are searched for; and all that can be done, is to form conclusions from the nature of the remote causes; all the known predisponent causes of tetanus, epilepsy, hysteria, and simple convulsion, are also predisponent causes of this disease. The canine poison is often insufficient to produce the disease without the co-operation of these causes.

19. The dreadful symptom which has gained this disease a separate discussion from any other convulsive affection, is now generally allowed to be satisfactorily explained on the association of ideas; the sight of water, or sound produced by any fluid is explicable in the same manner; why all the external senses are so easily affected in this disease, is easily explained from the increased irritability of the

L 2 whole

* The appearance and progress of the symptoms of Rabies in the dog are very generally known.

† Convulsion and spasm sometimes take place from an opposite state of the muscular fibre, such as is produced by lead; calomel useful in the latter, not in the former.

whole body producing morbid action, and sensation from the application of healthy stimuli. Solids are generally swallowed with more ease than fluids, during the presence of the symptom of hydrophobia, because an affection of the muscles of deglutition, proceeding from the disease under consideration, differs most evidently from an affection of the same muscles proceeding from a state of inflammation, and consequently distension. In the latter, liquids are swallowed with greater ease, because they require less exertion of the muscles affected than solids, the swallowing of which gives great pain, by increasing the preternatural distension already existing; in the former, liquids are swallowed with greater difficulty than solids, because they require a greater exertion of the muscles, over which the patient has entirely lost the power of command; but solids are enabled to descend by their own bulk, proving a resistance to the spasms from the power of distension, which liquids do not possess in the same degree.

20. The disease produced by the poison of a rabid animal seems very properly placed by nosological writers as a genus in the order of Spasm, and class of Neuroses; but, on account of the aquæ pavor being a symptom of other diseases, and the disease under consideration having existed without that symptom, and from the wish, which all should feel, of art not multiplying diseases, by exhibiting more genera than requisite, we may venture no longer to distinguish this disease by the name of a symptom, but placing it under the genus Convulsion, as a species, give the following definition of it. *Convulsion, from the poison of a rabid animal, almost always accompanied with a dread of liquids.* All those names by which this disease has been distinguished, that give any idea of the presence of mania, in any sense of the word, are founded in error. We may with equal propriety apply the term rabies to the delirium in typhus, and many other diseases. It appears to be placed with more propriety as a species of convulsion, than as a species of the genus Tetanus, because spasmodic diseases have been always divided into two kinds; the one, as in tetanus, where the morbid contraction is not followed by relaxation; the other, as in convulsion, where the morbid contraction is followed by spontaneous relaxation.

*Cure of Convulsion from the Poison of a Rabid Animal.*

When superstition, fable, prejudice, and unusual dread at the appearance of this disease are banished from the minds of medical men, and a just theory shall lead the way to to a steady practice, and when we consider that the variolous and venereal contagions, which threatened to destroy half the human race, have by means of inoculation* and dilution, by mercury and the nitric acid, been rendered by degrees less and less formidable, we may hope also to see the effects of the canine poison gradually counteracted or removed; and the medical world receive the disease amongst the number that can be combated by the powers of art and medicine. Perhaps this view of the disease, however simplified, by considering it as a convulsive affection, will be thought by some, not to give much more hope from such powers, since tetanus, epilepsy, and other convulsive affections, but tetanus in particular, to which this disease has the nearest resemblance, is commonly fatal. But there must be allowed to be at all times a better ground for hope, when the means of cure are conducted with firmness and perseverance, the result of a rational theory, than when doubt, timidity, mystery, and empiricism, direct our practice. It would be in vain to point out the numerous instances of these being only the guides of practice, in the prevention and cure of this disease, as it would be to point out the efforts which they have dictated at different times. The burned crabs of Aeschrion; Dessault's pancake of oyster-shells; the lichen cinereus terrestris of Palmerius and Dr. Mead; the turbith mineral of Dr. James, with the Piator medicine, and others, have all met with the fate they deserved. The Tonquin medicine, consisting of native and fictitious cinnabar and musk, introduced by Sir George Cobb, survived a little longer, and acquired a fame nearly as great as the Ormskirk medicine, prepared by the late Mr. Hill. Both of these proposed remedies have built the little fame they have obtained, on the circumstance of only one of a great number bitten by a rabid animal being seized with the disease; but one positive proof of their failure, and there are two many in existence, is worth a hundred negative proofs of their success.

Dr. Heysham and Professor Black have analysed the Ormskirk medicine, and found it to consist of half an ounce of powdered chalk, ten grains of alum, three drachms of armoniac bole, one drachm of the powder of elecampane root, and six drops of oil of anise. On such a composition what hopes are to be placed? Amongst the remedies that have proved equally fallacious, blood-letting

* Written previous to the discovery of the cow-pox.

and the warm bath may be named. There is no instance of success, where either of these means were used. Mercury has experienced the most complete trials; but the evidence in favour of it, is no better than that in favour of the Ormskirk medicine. Leaving these all behind, let us turn our attention to the plan of prevention and treatment, which has gained the most general approbation.

As a means of prevention, the method of dilution, pointed out by Dr. Haygarth, deserves our first attention; he recommends to wipe the wound with a dry cloth, so as to absorb all moisture, then abundantly, and with the most persevering attention, to wash the part with water quite cold for several hours. After this warm water is to be used, to produce a flow of blood, which is to be poured from the spout of a tea-kettle held up at a considerable distance. The ablution should be accomplished with great diligence, and without delay.* In a bad wound with much laceration, to this ablution, cupping and syringing are to be added; and in addition to this, it has been proposed that the wound should be enlarged, and even excision of the lacerated parts, when circumstances will admit of it. Dr. Percival has proposed, as a farther security, that the parts after ablution may be washed with the gastric liquor of an animal recently killed, or with the juice of rennet;† next to ablution, *the keeping up a free discharge from the wound for a great length of time, seems to be of the greatest importance.* This may be effected by repeated blistering, or by escharotics. It may here be remarked, that all trials to arrest the poison by means of caustics, gunpowder, and the like, have invariably failed; but after ablution their use to keep the wound open may be of service. Ligatures above and below the wounded part, where they can be applied, have been recommended during the ablution, by Dr. Percival. Apparently, simple means produce very salutary effects; perhaps, after the ablution recommended by Dr. Haygarth, the pouring of warm cow's milk from a tea-kettle in the same manner upon the wound, may be of use; we know that milk has the power of counteracting the effects of some poisons received into the stomach. Such are

---

* The effect of dilution in rendering the contagion of fever, small-pox, &c. inert, gives great reason to hope the happiest consequences from this plan.

† When parts about the mouth are wounded, washing them well with the same, or with sea water, or a weak solution of the vegetable alkali has been recommended.

are the means of prevention most likely to succeed, we now come to the treatment of the disease when such have not been used, or shall unfortunately fail. And here I may observe, that I was of late more immediately led to the consideration of the cure of this disease by seeing Dr. Arnold's very interesting case of Hannah Springthorpe, which was treated by stimulants and antispasmodics, and terminated favorably. From this case and some others, it appears that our reliance ought to be entirely on such remedies, when the symptoms of the disease appear. Opium, musk, cuprum ammoniacum, calcined zinc, with the cold bath, have been particularly named as most adapted to the disease. Doctor Percival thinks that the digitalis, from its quick action and sedative powers, seems to promise to be of service in this disease. Dr. Shadwell's case of John Cumbus, a drover, seems to shew, that the internal and external use of oil is of considerable service in allaying the irritability and spasms. From our knowledge of its component parts, it promises to be useful to answer this indication; but instead of frictions, perhaps a frequent immersion of the whole body in oil of a temperature a little above that of the body, might be more efficacious. The warm bath has always given momentary relief, which I attribute to the stimulus of heat; but the effect of this stimulus, applied through the medium of water, may not be so permanent as when applied through the medium of oil; besides the oil itself may act as a stimulant, and its effects may be also permanent. The internal use of oil should also be had recourse to; and when its use in this way is prevented by the spasmodic affection of the muscles of deglutition, perhaps it may be conveyed into the stomach by the same means* as have been recommended for throwing food into the stomach in a paralysis of those muscles.

The benefit experienced from the use of the spirit of caustic volatile alkali, in preventing the bad effects of the bite

---

* A fresh eel skin, of rather a small size, (or the intestine of some animal will probably answer as well) drawn over a probang, and tied up at the end where it covers the sponge, and tied again close to the sponge where it is fastened to the whalebone, and a small longitudinal slit made into it immediately above this upper ligature. To the other end of the eel skin a bladder with a wooden pipe is fixed, the tube being large enough to permit the end of the probang to pass into the bladder without filling up the passage. The probang, thus covered, is introduced into the stomach, and food or medicines are put into the bladder, and squeezed down thro' the eel skin.

bite of a species of viper, induces Dr. Bardsley to think, that this medicine may be of use in this disease. We cannot have too large a magazine of powerful stimulants to resort to in such a state of the body, as a succession is required in a rapid manner to produce any sensible effect in so short a period as commonly is allowed for their action; perhaps the *Peruvian balsam* may be no trifling vehicle for the *volatile alkali.* Opium, that anchor of all our hopes in many diseases, is to be chiefly depended on in this disease. The late Mr. Hill used to give it with his medicine; but as a preventive, it cannot be of any use. I remember that Dr. Black, in his lectures, always mentioned his suspicion of arsenic forming a part of Mr. H's remedies. From the powerful tonic effect of the *mineral solution,* it appears adapted to every indication in the cure of convulsions; I have stopped long established epileptic paroxysms by its powers. It has not been mentioned by any author I have read on this disease.*

Thus I have endeavoured to comprise, within narrow limits, the means of prevention and cure of this yet terrific disease; and I cannot conclude without expressing my ardent hope, that by such well regulated methods the disease will in time be overcome.

J. WOOD.

*July* 4, 1797.

---

To the above paper I will only now add, that next to dilution, to which no objection can be made, the keeping the wound open for a proper time appears to be of the first importance, and that *tonics* and *antispasmodics* are chiefly to be relied upon if the symptoms of the disease should take place. Perhaps the operation of the *arsenical solution* may be too slow in such a rapid disease, after the symptoms have appeared, and that the *cinchona* with *valerian* may have a more immediate effect. I believe I may venture to assert, that few practitioners in the North have opportunities of seeing such a variety of spasmodic diseases as falls to my situation to observe; and I can say, confidently, that in some the *rad. valer. sylv.* is of great use; and that in simple convulsion I have derived most decided relief from asafœtida, in form of solution, which I give

* Since the date of this, I think arsenic has been recommended in your Journal, and by Dr. Bardsley, if I recollect right.

I give as an Enema also, when requisite.—To all the means of cure in Hydrophobia may be added *topical* applications to the throat, which in one instance, it seems, was of the greatest use, and with the particulars of which I will now conclude. I think the case did find its way, some time ago, into a periodical work, and I believe by means of the son of the Author; yet as many may not have seen it, I will here repeat the leading circumstances of it; they are from a MS. of the late Dr. Turnbull.

Robert Dixon, a weaver, of Norham Mains, near Berwick, was bitten on the leg by a mad dog, 30 July, 1761. The symptoms of Hydrophobia soon appeared; pain gradually ascended from the wound to the knee, thigh, stomach, with sickness and oppression at the breast. These sensations daily increased, and were followed by convulsions and strictures in the throat, which threatened suffocation, particularly when water was presented to him. To the wounded part a caustic was applied, and it was kept open by blistering, and stimulating ointment, *from the first,* until some time after all the symptoms were entirely gone. The leg was often bathed with warm oil. A teaspoonful of a tonic electuary was given four times a day, consisting of bark, valerian, musk, and camphor; opium was also given in large doses, to assuage the irritation and spasms. To the throat was applied a plaister, consisting of opium, frankincense, camphor, asafœtida, and gum galbanum. The man, after his recovery, declared that he *felt* more relief from *the plaister* than any other thing; he said that it gave a pleasant warmth to his throat, and from thence its effects followed in the same direction to the wound as the pain had ascended from it.

*Newcastle, Dec.* 12, 1808.

CRITICAL

